# Safety Evaluation of Turmeric Polysaccharide Extract: Assessment of Mutagenicity and Acute Oral Toxicity

**DOI:** 10.1155/2013/158348

**Published:** 2013-12-17

**Authors:** Chandrasekaran Chinampudur Velusami, Srinivasa Rao Boddapati, Srikanth Hongasandra Srinivasa, Edwin Jothie Richard, Joshua Allan Joseph, Murali Balasubramanian, Amit Agarwal

**Affiliations:** R&D Centre, Natural Remedies Private Limited, 5B, Veerasandra Indl. Area, 19th K. M. Stone, Hosur Road, Electronic City Post, Bangalore 560100, Karnataka, India

## Abstract

*Curcuma longa* Linn. (Zingiberaceae) commonly known as turmeric has long been used for centuries as a spice and household remedy. The present study was carried out to assess the possible mutagenic potential and acute oral toxicity of polysaccharide extract of turmeric rhizome (NR-INF-02) using standard tests. The standard battery of *in vitro* genotoxicity tests, bacterial reverse mutation test (BRMT), chromosome aberration (CA), and micronucleus (MN) tests were employed to assess the possible mutagenic activity of NR-INF-02 (Turmacin). The results showed no mutagenic effect with NR-INF-02 up to a dose of 5000 *µ*g/mL in BRMT. The results on CA and MN tests revealed the non clastogenic activity of NR-INF-02 in a dose range of 250.36 to 2500 *µ*g/mL with and without metabolic activation (S9). In acute oral toxicity study, NR-INF-02 was found to be safe up to 5 g/kg body weight in Wistar rats. Overall, results indicated that polysaccharide extract of *C. longa* was found to be genotoxically safe and also exhibited maximum tolerable dose of more than 5 g/kg rat body weight.

## 1. Introduction

Turmeric (*Curcuma longa*) has been used for centuries in Ayurvedic medicine, which amalgamate the medicinal goods of herbs with food. This astonishing herb has established its way into the attention in the west because of its wide range of medicinal benefits [1]. In *ayurveda*, rhizome of turmeric is used as medicines against skin, gastrointestinal, respiratory, hepatic, and biliary disorders [2]. The active constituents of *C. longa* are the flavonoid curcumin (diferuloylmethane) and various volatile oils, including tumerone, atlantone, and zingiberene. Other constituents include sugars, proteins, and resins. The best researched active constituent is curcumin, which comprises 0.3–5.4 percent of raw turmeric [1]. Components of turmeric especially curcumin has been shown to have anti-inflammatory, antiviral, and anticancer properties [3–5]. Among the phytoconstituents of *C. longa*, curcuminoids are considered as an important active molecule and also exhibited wide range of pharmacological activities. The literature review confirmed plethora of information available on safety aspects of curcumin and essential oil fractions of *C. longa* [3, 6–8].

While curcuminoids based extract were well studied for their pharmacological and safety aspects, polysaccharide extract of *C. longa* is gaining importance since it showed to have various pharmacological activities, which include antidiabetic, antitumour, antidepressant, antioxidant, antimicrobial, antifertility, hepatoprotective, and immunomodulatory properties [9–16]. NR-INF-02, a polysaccharide extract prepared from rhizome of *C. longa* had shown clinical efficacy in a randomized placebo controlled study on 120 human patients (37 males and 83 females) affected with primary osteoarthritis [17]. NR-INF-02 deserved as an effective option for the treatment of patients with primary painful knee and joint pains and also reduced the need of analgesics as a rescue medication [17]. Also, NR-INF-02 showed immune-stimulatory and anti-inflammatory effects in *in vitro* models by influencing various cytokines involved in immune regulation [18]. Similarly, the immunostimulatory effects of *C. longa* polysaccharides on peripheral blood mononuclear cells was investigated and the findings revealed the potential use of *C. longa* polysaccharide extract as an adjuvant supplement for cancer patients, whose immune activities were suppressed during chemotherapies [16].

Polysaccharide extract of *C. longa* needs evaluation for their safety due to its growing demand on reported preclinical therapeutic indications. Also, before setting of a clinical trial of an herbal product, its safety must be evaluated by toxicity test procedures. Also, this toxicity evaluation is helpful for the estimation of an initial safe starting dose and dose range for the human trials and the identification of parameters for clinical monitoring for potential adverse effects. Hence, the goal of the present investigation is to characterize the toxicological profile of polysaccharide extract from *C. longa* rhizome (NR-INF-02).

In order to evaluate toxicological profile, the ability of NR-INF-02 to induce mutations was assessed in indicator microorganisms using bacterial reverse mutation test. The effect of NR-INF-02 on the genetic system was weighed up by analyzing induced chromosomal aberrations and micronucleus in mammalian cells. An acute oral toxicity study was performed after single oral dose of NR-INF-02 to determine immediate toxic effect. All these tests were conducted based on recommendation of the OECD guidelines [19–22].

## 2. Materials and Methods

### 2.1. Collection and Identification of *Curcuma longa*

The rhizomes of *Curcuma longa* Linn. were collected from different parts of Tamil Nadu State, India and authenticated at NISCAIR (National Institute of Science Communication and Information Resources). A voucher specimen (no. 653) was deposited in our herbarium.

### 2.2. Preparation of Polysaccharide Extract of *Curcuma longa* Extract (NR-INF-02)

NR-INF-02 is manufactured and registered as Turmacin by Natural Remedies Pvt. Ltd., Bangalore, India. Coarsely powdered rhizomes of *C. longa* were subjected to steam distillation and the turmeric oil was separated and collected. The rhizomes were further extracted by refluxing with water in a commercial extraction facility. The liquid water extract was concentrated by distillation under vacuum and the resultant concentrated liquid was spray dried to obtain a free flowing powder. NR-INF-02 was prepared by blending 99 parts of spray dried water extract of *C. longa* and 1 part of the turmeric oil followed by sieving. Yield of this extract is 10.5% w/w.

Details about the preparation and characterization of NR-INF-02 were described as earlier [18]. In brief, NR-INF-02 was standardized to contain polysaccharides (>10% w/w) by high performance liquid chromatography [23]. As reported previously, NR-INF-02 was found to contain negligible amount of curcumin [18]. NR-INF-02 was assessed for presence and absence of microbial (total aerobic microbial count, total yeast and mould count, bile tolerant gram negative bacteria, *E. coli*, Salmonella species, *S. aureus*), heavy metals (lead, arsenic, cadmium, and mercury), pesticides (75 different pesticides), and aflatoxin levels as per the method described by the United States Pharmacopeia [24]. Results indicated that NR-INF-02 is compliant to the limit set by United States Pharmacopeia (USP) and British Pharmacopeia [24, 25]. Stability of NR-INF-02 was assessed using accelerated and real time protocols of ICH guidelines [26]. NR-INF-02 did not undergo any major changes (physically, chemically, and microbiologically) when stored under accelerated and real time condition for 6 months and 12 months, respectively. Based on the stability study it is recommended to store NR-INF-02 below 30°C in an airtight container.

### 2.3. Source of Chemicals

2-aminoanthracene (2-AA), benzo[a]pyrene, colchicine, cytochalasin B, D-glucose-6-phosphate, 3-(4,5Dimethylthiazol-2-yl)-2,5-diphenyltetrazolium bromide, nicotinamide adenine dinucleotide phosphate, mitomycin C, 2-nitrofluorene (2-NF), 4-nitroquinolene-N-oxide (4-NQO), phytohemagglutinin, potassium chloride (KCl) were purchased from Sigma-Aldrich. Fetal bovine serum (FBS) was procured from Hyclone. Aroclor 1254 induced S9 fraction was obtained from Moltox. The Ames II Automated System for High Throughput Screening kit containing Salmonella strains like TA98 strain and TAMix strain, ampicillin, exposure medium incubation bag, indicator medium and growth medium were purchased from Xenometrix. Ham's F12K medium, RPMI 1640 medium and Trypsin-EDTA were purchased from Gibco Life Technologies.

### 2.4. Bacterial Strains and Mammalian Cells

The bacterial reverse mutation test (Ames II) was performed using *Salmonella typhimurium *histidine auxotrophs, TA98 strain and TAMix strain. Genotyping on Salmonella strains like TA98 strain and TAMix strain was confirmed before conducting the mutagenicity study. Concentration of standard mutagen(s), S9 and incubation time were optimized and selected based on previous publications [27–31] which we have followed in the current genotoxicity study. 10 **μ**L of freshly thawed frozen Salmonella strains like TA98 strain and TAMix strain were inoculated to 10 mL of growth medium and the cultures were grown overnight at 37°C in an environmental shaker set at 180 rpm in the presence of ampicillin (50 **μ**g/mL). The human peripheral blood for chromosome aberration study was collected from healthy human volunteers. Lymphocytes of human peripheral blood cells were cultured in RPMI 1640 media supplemented with 20% heat-inactivated fetal bovine serum (FBS) and 2% phytohemagglutinin-M for 48 h in culture tubes fitted with loose caps at 37°C in a humidified atmosphere of 5% CO_2_. Chinese Hamster Ovary cells (CHO-K1) for micronucleus test was procured from American Type Culture Collection. CHO-K1 cells were cultured in Ham's F12K media supplemented with 10% FBS, in 37°C incubator maintained at 5% CO_2_.

### 2.5. Bacterial Reverse Mutation Test

The bacterial reverse mutation test (Ames II) was used to identify the ability of test substance to induce reverse mutation at histidine loci in Salmonella strains like TA98 strain and TAMix strain (mixture of six base pair mutant strains TA7001-7006). This mutagenicity test was performed according to standard procedure [19, 27–32]. Briefly, *Salmonella typhimurium *tester strains like TA98 strain and TAMix strain were exposed to NR-INF-02 by fluctuation method with and without S9. Dulbecco's Phosphate Buffered Saline (DPBS) was used to solubilise NR-INF-02 and also designated as a vehicle control. NR-INF-02 at a maximum concentration of 5000 **μ**g/mL and subsequent concentrations, 1582.28, 500.72, 158.46, 50.14, and 15.87 **μ**g/mL were selected to assess the mutagenic effect. NR-INF-02 at concentration range of 15.87 to 5000 **μ**g/mL, Salmonella tester strain like TA98/TAMix strain with and without S9 mix were added along with reversion indicator media into the wells of 384-well plates and incubated for 48 h at 37°C. All doses of the NR-INF-02, vehicle control (DPBS), and positive controls were plated in triplicates; 2-NF (2 **μ**g/mL) + 4-NQO (0.5 **μ**g/mL) and 2-AA (5 **μ**g/mL) were used as positive controls in the absence and presence of S9 respectively. After incubation, the revertant colonies were counted and positive response was determined by significant increase in the mean revertant per plate of atleast one of the tester strains as compared to vehicle control.

### 2.6. Chromosome Aberration Assay Using Human Peripheral Blood Lymphocytes

Chromosome aberration test was performed as per OECD guideline no. 473 [20]. Human blood was collected aseptically from one healthy, non-smoking male donor. 0.5 mL of blood, 9.5 mL growth medium (RPMI 1640 + 20% heat inactivated fetal bovine serum) were added and cultured using phytohemagglutinin-M (2% v/v) for 48 h at 37°C in a humidified atmosphere of 5% CO_2_ in air. The cultures were gently shaken daily. After 48 h, cells were treated with NR-INF-02 (250.35, 791.14, 2500 **μ**g/mL) in presence and absence of S9. NR-INF-02 at a maximum concentration of 2500 **μ**g/mL was used since it did not produce evident cytotoxicity to human lymphocytes. RPMI 1640 medium was used to dissolve NR-INF-02 and also designated as vehicle control. Mitomycin C (0.2 **μ**g/mL) was used as positive control for both short term (4 h) and long term (36 h) exposure studies in the absence of S9, whereas, benzo[a]pyrene (20 **μ**g/mL) was used as positive control for short term exposure (4 h) study only with S9. After short term exposure, the treatment medium was removed by centrifugation and cultures replenished with growth medium (RPMI 1640 + 10% FBS). The cells were again returned to the incubator to complete 1.5 cell cycle lengths. For long term, the cells are treated with test compound for the entire 1.5 cell cycle length. Cells were exposed to colchicine (0.3 **μ**g/mL) for 3 h prior to harvest. After 3 h, cells were centrifuged, resuspended in hypotonic medium (75 mM KCl) and fixed (prechilled, 3 parts of ethanol and 1 part of glacial acetic acid). Approximately 3-4 drops of the fixed cell suspension from the height of 30 cm was dropped onto a clean microscope slide and stained with 10% Giemsa solution for 10 min. Two hundred metaphase chromosome spreads per treatment were examined under the microscope for chromosome break, chromatid break, deletion, ring, dicentric and rearrangements as indicated by Savage [33]. Only metaphases containing 46 ± 2 centromeres (chromosomes) were considered for analysis. The number of cells with aberrations and the number of aberrations were recorded. Experiments were performed in duplicates per treatment. Cytotoxicity was determined by calculating Mitotic index (MI) according to the formula

(1) $$\begin{array}{c} \text{percent}\;\text{MI}=\left(\frac{\text{no}.\;\text{of}\;\text{cells}\;\text{in}\;\text{metaphase}}{\text{total}\;\text{number}\;\text{of}\;\text{cells}}\right)\times 100. \end{array}$$

### 2.7. *In Vitro* Micronucleus Test in Chinese Hamster Ovary (CHO-K1) Cells

Micronucleus test was carried out according to the OECD guideline no. 487 [21]. CHO-K1 cells were incubated with NR-INF-02 (250.36 **μ**g/mL, 791.14 **μ**g/mL and 2500 **μ**g/mL) in both short (4 h) and long term (18 h) exposure studies with and without S9. Ham's F12K media was used to solubilise NR-INF-02 and also designated as a vehicle control. Mitomycin C (0.2 **μ**g/mL) was used as positive control in the absence of S9 for both short and long term exposure studies. Benzo[a]pyrene (20 **μ**g/mL), was used only with S9 in a short term exposure study. After short term exposure, treatment medium was removed by aspiration and the cells were rinsed with Hank's balanced salt solution (HBSS) and replenished with complete Ham's F12 medium containing cytochalasin B (3 **μ**g/mL) to arrest cytoplasmic division of the cells. The cells were then returned to the incubator for an additional 18 h. For long term study, NR-INF-02 and cytochalasin B were added together and incubated for 18 h. After trypsinization, cells were suspended in 75 mM KCl and fixed using precooled fixative [ethanol and glacial acetic acid (3 : 1)]. Approximately 3-4 drops of the cell suspension were dropped from the height of 5 cm onto a clean microscope slide. The slides were air dried overnight and stained with 10% Giemsa for 10 min. Experiments were performed in duplicates. The criteria for selecting the binucleated cells were based on the report by Fenech [34]. Per treatment, 2000 cytoplasm division arrested cells were examined for the presence of micronucleus. Cytokinesis Block Proliferation Index (CBPI) was determined to calculate the cell toxic effect of treatment according to the formula

(2) CPBI=(no.  of  mononucleated  cells+2(no.  of  binucleated  cells)+3(no.  of  multinucleate  cells))×(total  number  of  cells)−1.

### 2.8. Acute Oral Toxicity

The animal experiment was conducted according to the CPCSEA (Committee for the Purpose of Control and Supervision of Experiments on Animals) guidelines and after approval by the Institutional Animal Ethics Committee (IAEC). Female albino Wistar rats (8–12 weeks) were accommodated in polypropylene cages and temperature was maintained between 25 ± 2°C with 12 h each of dark and light cycle. The rats were fed with standard laboratory pelleted feed (M/s Gold Mohur Foods & Feeds Ltd., Bangalore, India). The rats were fasted overnight before and 3 h after the administration of NR-INF-02. Acute oral toxicity study was performed as per the OECD Guideline for the Testing of Chemicals (Test no. 420, Section 4: Health Effects) Acute Oral Toxicity—Fixed Dose Procedure [22]. NR-INF-02 solubilized in demineralized water was administered by oral route to rats at the limit dose of 5 g/kg body weight in a sequential manner. On the day of dosing, all the animals were observed for mortality and clinical signs for first 10 min, 30 min, 1 h, 2 h, 4 h, and 6 h after dosing and thereafter twice daily for mortality and once a day for clinical signs, for 14 days. Animals were sacrificed at the end of the study period of 14 days.

### 2.9. Statistical Analysis

Data are expressed as mean ± standard deviation (SD). One way analysis of variance (ANOVA) was performed on the results followed by a Dunnett's test for multiple comparisons using GraphPad Prism 5.0 (GraphPad Software, Inc., San Diego, CA) statistical software package. The significance level was chosen at *P* < 0.05 for all statistical analyses in comparison to the respective vehicle control.

## 3. Results

### 3.1. Bacterial Reverse Mutation Test

To detect possible point mutations by NR-INF-02, histidine requiring mutants of *S. typhimurium *strains likeTA98 strain and TAMix strain, with and without S9 were used. No significant bacterial cell toxicity was observed after treatment with NR-INF-02 up to a maximum concentration of 5000 *μ*g/mL. The occurrence of spontaneous reversion for TA98 strain and TAMix strain is in agreement with previous reports [27–31]. ANOVA followed by Dunnett's multiple comparison tests indicated that treatment of NR-INF-02 at concentrations, namely, 15.87 **μ**g/mL, 50.14 **μ**g/mL, 158.46 **μ**g/mL, 500.72 **μ**g/mL, 1582.28 **μ**g/mL, and 5000 **μ**g/mL, did not show any significant increase in the number of revertant colonies in both Salmonella strains like TA98 strain and TAMix strain with and without S9 when compared to vehicle control. Positive controls, 2-NF + 4-NQO, 2-AA demonstrated a significant (*P* < 0.05) increase in the number of revertant colonies in the absence and presence of S9 respectively (Tables 1 and 2). These results confirmed the nonmutagenic property of NR-INF-02 in TA98 strain and TAMix strain of *S. typhimurium.*

### 3.2. *In Vitro* Chromosome Aberration Analysis in Human Peripheral Blood Lymphocytes

Mitotic index values indicated that NR-INF-02 at dose level, namely, 250.36 **μ**g/mL, 791.14 **μ**g/mL, and 2500 **μ**g/mL, did not produce cell toxicity in both short term (4 h) and long term exposure (36 h) with and without S9 (Tables 3, 4, and 5). Treated cells arrested at metaphase stage were analyzed for possible structural chromosomal aberrations. Exposure of NR-INF-02 at the indicated concentrations (250.36 **μ**g/mL, 791.14 **μ**g/mL and 2500.0 **μ**g/mL) to human lymphocytes did not induce a statistically significant increase in the number of cells with chromosome aberrations in the absence and presence of S9 in both short and long term exposure studies. As determined by ANOVA followed by Dunnett's multiple comparison tests, vehicle control cultures had an insignificant number of structural chromosomal aberrations which were within the limit of published data [28, 30, 31]. The positive controls, mitomycin C, and benzo[a]pyrene were found to induce statistically significant (*P* < 0.05) number of structural chromosomal aberrations, namely, chromosome break, chromatid break, deletion in absence and presence of S9 respectively in human peripheral blood lymphocytes (Tables 3, 4 and 5).

### 3.3. *In Vitro* Micronucleus Analysis in CHO-K1 Cells

Statistical analysis (ANOVA followed by Dunnett's tests) indicated that CBPI values did not differ significantly between the tested concentrations of NR-INF-02 and vehicle control. Mitomycin C treatment showed significant (*P* < 0.05) reduction on viability of CHO-K1 cells in both short term and long term exposure cultures as evident from decrease of CBPI values in comparison with vehicle control. In the micronucleus test, no statistically significant increase in the frequency of cells with micronucleus was observed upon treatment with NR-INF-02 at the indicated concentrations (250.36 **μ**g/mL, 791.14 **μ**g/mL and 2500 **μ**g/mL), in the presence and absence of S9, in both short (4 h) and long term (18 h) exposure cultures. Vehicle control had an insignificant number of micronucleus which were within the limit of published data [28, 30, 31]. As evident, the positive controls mitomycin C and benzo[a]pyrene formed significant (*P* < 0.05) increase in the frequency of cells with micronucleus in absence and presence of S9 respectively. Percent micronucleated, binucleated cells, and CBPI values for all treatment cultures are presented in Table 6.

### 3.4. Acute Oral Toxicity

NR-INF-02 was evaluated for its acute oral toxicity by administering as a single oral dose to albino Wistar rats. NR-INF-02 was administered orally in a sequential manner to five rats at the limit dose level of 5000 mg/kg body weight. On the day of dosing, all the animals were observed for mortality and clinical signs for first 10 min, 30 min, 1 h, 2 h, 4 h, and 6 h after dosing and thereafter twice daily for mortality and once a day for clinical signs, for 14 days. The body weight of rats was recorded and weekly body weight gain was calculated. After the observation period of 14 days, all surviving rats were sacrificed and subjected to complete necropsy. The treated rats did not show any adverse clinical signs immediately following dosing and during the observation period of 14 days. In sighting and main studies, treatment with NR-INF-02 did not reveal any adverse effects on the body weight gain at first and second week of observation. Overall, the percent body weight gain during the complete 14 days observation period was found to be normal in all the treated animals. On necropsy, no major gross pathological changes were observed in NR-INF-02 treated rats (Tables 7 and 8). Based on the findings of the present study, NR-INF-02 was found to be safe after oral administration as a single dose of 5000 mg/kg to female albino Wistar rats.

## 4. Discussion

This study is focused on characterization of toxicological properties of *C. longa* polysaccharide extract: mutagenicity, clastogenicity, and acute oral toxicity. These evaluations are a fundamental part of the clinical study aimed at determining the risk/benefit ratio of its use in the field of human health and subsequently introducing this extract into dietary supplement practice. Hence, this study was conducted to investigate the possible genotoxic potential of NR-INF-02 using bacterial reverse mutation, chromosomal aberration and micronucleus tests.

The significance of the bacterial reverse mutation test has been clearly confirmed as a suitable primary test for the detection of potential mutagens and carcinogens, and since midseventies this assay has been routinely used as a screening assay to predict carcinogens [32]. NR-INF-02 at dose range of 15.87–5000 *μ*g/mL did not induce any significant increase in the revertant colonies both in the presence and absence of metabolic activation. These results confirmed the nonmutagenic activity in Salmonella strains like TA98 strain and TAMix strain. This is in agreement with the absence of genotoxicity of *C. longa* extracts tested *in vitro* by several researchers [6, 35–38]. Moreover, a substantial lack of genotoxic activity is reported in the literature for extracts obtained from polysaccharide fraction of other species of the genus Curcuma, such as *C. zedoaria* [39]. Apart from lack of mutagenicity, antimutagenic property of *C. longa* extracts against several classical mutagens were reported [35, 40].

The study of DNA damage at the chromosomal level and micronuclei formation is a vital part of genetic toxicity screening [20, 21]. Chromosome aberration and micronucleus tests were carried out with NR-INF-02 using peripheral blood lymphocytes and CHO-K1 cells, respectively. The effect of NR-INF-02 on dividing cell population was examined by studying mitotic index and CBPI in peripheral blood lymphocytes and CHO-K1 cells, respectively. The mitotic index is used to quantify differences in cell division when an environmental parameter is changed [20]. CBPI indicates the number of cell cycles per cell during the period of exposure to cytochalasin B [21]. Treatment effect of NR-INF-02 on MI and CBPI indicated that cells did not differ in their capability to divide in comparison to vehicle control. Also, NR-INF-02 did not induce significant chromosome aberrations and micronuclei formation in mammalian cells. These results are in accordance with the absence of clastogenic effect of *C. longa* and *C. zedoaria* extracts tested and reported previously [39–42]. In addition, anticlatsogenic effect of *C. longa* was observed against benzo (a) pyrene induced micronucleus in mice and these results indicated that components of *C. longa* could help in cancer chemoprevention [35, 40].

Acute oral toxicity data on NR-INF-02 is used to satisfy hazard classification and labelling requirements for its risk assessment in human health and environment [43]. In the present study, acute oral toxicity assessment of NR-INF-02 did not cause mortality, abnormal clinical signs or any significant pathological changes at a dose level of 5000 mg/kg body weight. Also, the overall body weight gain was found to be normal in all the treated rats and hence resulted in labelling the NR-INF-02 as unclassified in the hazard category according to Globally Harmonised System [22]. Current acute oral toxicity study results are in agreement with previous study which demonstrated no toxic effects upon ingestion of turmeric extracts by rats [44].

In contrary, curcumin induced significant increases in sister chromatid exchanges and chromosomal aberrations in cultured Chinese hamster ovary cells [36]. In addition, hepatotoxicity was observed in rodents fed turmeric for chronic duration [45, 46]. National Toxicology Program conducted detailed two year safety study; results indicated an equivocal evidence of carcinogenic activity, increased incidences of ulcers, hyperplasia, and inflammation of the forestomach, cecum, and colon in rats of turmeric oleoresin exposed groups [36]. Differences on results observed between the current study and published results might be due to variation in phytochemical composition of test items.

NR-INF-02 is a polysaccharide containing extract and is a complex mixture of natural substances. As evident in our results, NR-INF-02 contains acceptable pharmacopeial limits for microbes, heavy metals, pesticides, and aflatoxin contents which further confirm the quality assurance of this extract.

The use of genotoxicity testing is to determine *C. longa* extract influences genetic material or may cause cancer. The results confirmed the genotoxic safety of *C. longa* extract in a battery of genotoxicity tests. Acute oral toxicity data on *C. longa* extract is used to satisfy hazard classification and labelling requirements for its risk assessment in human health and environment [43]. In the present study, acute oral toxicity assessment of *C. longa* extract did not cause mortality, abnormal clinical signs, or any significant pathological changes upto the dose level of 5000 mg/kg body weight. Also, the overall body weight gain was found to be normal in all the treated rats and hence resulted in labelling the *C. longa* extract as unclassified in the hazard category according to Globally Harmonised System [22]. Also, clinical effective dose of NR-INF-02 for pain management in human osteoarthritis patients was achieved at 1 g/day with no adverse effects after daily oral intake of 42 days [17].

## 5. Conclusion

In conclusion, polysaccharide extract of *C. longa* was found to be non-mutagenic to *S. typhimurium* strains like TA98 strain and TAMix strain. *C. longa* did not increase the occurrence of structural chromosomal aberrations in human peripheral blood lymphocytes and micronucleus formation in CHO-K1 cells. Also, it was found to be safe after oral administration as a single dose to female albino Wistar rats up to 5 g/kg body weight. Therefore, polysaccharide extract from rhizomes of *C. longa* is not mutagenic in the tested standard battery of genotoxicity tests and found to be safe in an acute oral toxicity study.

## Figures and Tables

**Table 1:** Mutagenicity testing of NR-INF-02 in *Salmonella typhimurium *TA98 strain.

Treatment	Concentration (*µ*g/mL)	Number of revertant colonies					
		S9	Individual colony counts			Mean ± S.D	Fold increase over baseline
			R1	R2	R3		
NR-INF-02	15.87	−	8	6	7	7.00 ± 1.00	0.83
		+	7	5	7	6.33 ± 1.15	0.90
	50.14	−	6	4	8	6.00 ± 2.00	0.71
		+	3	8	7	6.00 ± 2.65	0.86
	158.46	−	7	6	8	7.00 ± 1.00	0.83
		+	5	8	2	5.00 ± 3.00	0.71
	500.72	−	4	7	6	5.67 ± 1.53	0.67
		+	4	6	4	4.67 ± 1.15	0.67
	1582.28	−	8	8	8	8.00 ± 0.00	0.95
		+	3	7	4	4.67 ± 2.08	0.67
	5000.00	−	8	7	4	6.33 ± 2.08	0.75
		+	8	7	7	7.33 ± 0.58	1.05
Vehicle control	0	−	8	4	7	6.33 ± 2.08	—
		+	6	7	5	6.00 ± 1.00	—
Positive control 2-NF + 4-NQO	2.00 + 0.50	−	47	46	48	47.00 ± 1.00*	5.58
Positive control 2-AA	5.00	+	48	48	48	48.00 ± 0.00*	6.85

R: replicate; *P < 0.05; 2-NF: 2-nitrofluorene; 4-NQO: 4-nitroquinoline-N-oxide; 2-AA: 2-aminoanthracene.

**Table 2:** Mutagenicity testing of NR-INF-02 in *Salmonella typhimurium *TAMix strain.

Treatment	Concentration (*µ*g/mL)	Number of revertants colonies					
		S9	Individual colony counts			Mean ± S.D	Fold increase over baseline
			R1	R2	R3		
NR-INF-02	15.87	−	0	2	0	0.67 ± 1.15	0.67
		+	0	0	0	0.00 ± 0.00	0.00
	50.14	−	0	0	1	0.33 ± 0.58	0.33
		+	0	2	0	0.67 ± 1.15	0.37
	158.46	−	0	0	2	0.67 ± 1.15	0.67
		+	2	1	2	1.67 ± 0.58	0.92
	500.72	−	0	1	0	0.33 ± 0.58	0.33
		+	0	0	1	0.33 ± 0.58	0.18
	1582.28	−	2	1	1	1.33 ± 0.58	1.33
		+	0	2	0	0.67 ± 1.15	0.37
	5000.00	−	0	3	2	1.67 ± 1.53	1.67
		+	0	2	0	0.67 ± 1.15	0.37
Vehicle control	0	−	1	1	1	1.00 ± 0.00	—
		+	0	0	2	0.67 ± 1.15	—
Positive control 2-NF + 4-NQO	2.00 + 0.50	−	48	48	48	48.00 ± 0.00*	48.00
Positive control 2-AA	5.00	+	45	41	47	44.33 ± 3.06*	24.35

R: replicate; *P < 0.05; 2-NF: 2-nitrofluorene; 4-NQO: 4-nitroquinoline-N-oxide; 2-AA: 2-aminoanthracene.

**Table 3:** Clastogenicity study upon short term exposure (4 h) of NR-INF-02 in the absence of metabolic activation in human blood lymphocytes.

Treatment	Vehicle control		Positive control MMC (0.20 *µ*g/mL)		NR-INF-02 (2500.00 *µ*g/mL)		NR-INF-02 (791.14 *µ*g/mL)		NR-INF-02 (250.36 *µ*g/mL)	
	R1	R2	R1	R2	R1	R2	R1	R2	R1	R2
Total number of metaphase analyzed	100	100	100	100	100	100	100	100	100	100
Normal	99	100	92	92	98	97	98	96	98	100
Chromatid break	1	—	—	4	1	—	1	—	—	—
Chromosome break	—	—	—	1	—	1	—	—	—	—
Deletion	—	—	—	1	—	—	—	—	1	—
Ring	—	—	3	1	1	—	—	—	1	—
Dicentric	—	—	5	1	—	2	—	1	—	—
Fragment	—	—	—	—	—	—	—	1	—	—
Gap	—	—	—	—	—	—	1	1	—	—
Ploidy	—	—	—	—	—	—	—	1	—	—
Endoreduplication	—	—	—	—	—	—	—	—	—	—
Total aberrations	1	0	8	8	2	3	1	1	2	0
Total aberrations (Mean ± SD)	0.50 ± 0.70		8.00 ± 0.00*		2.50 ± 0.70		1.0 ± 0.00		1.00 ± 1.41	
Mitotic Index (Mean ± SD)	5.35 ± 0.49		2.63 ± 0.10*		3.75 ± 1.06		4.25 ± 0.35		4.0 ± 0.42	

R: replicate; *P < 0.05; MMC: mitomycin C; Ploidy, endoreduplication, gaps, and fragments are not considered as aberrations for calculations.

**Table 4:** Clastogenicity study upon short term exposure (4 h) of NR-INF-02 in the presence of metabolic activation in human blood lymphocytes.

Treatment	Vehicle control		Positive control B[a]P (20.00 *µ*g/mL)		NR-INF-02 (2500.00 *µ*g/mL)		NR-INF-02 (791.14 *µ*g/mL)		NR-INF-02 (250.36 *µ*g/mL)	
	R1	R2	R1	R2	R1	R2	R1	R2	R1	R2
Total number of metaphase analyzed	100	100	100	100	100	100	100	100	100	100
Normal	100	97	93	89	100	96	97	97	98	98
Chromatid break	—	—	3	2	—	—	—	—	—	1
Chromosome break	—	—	1	1	—	—	1	—	—	—
Deletion	—	—	—	—	—	—	1	—	—	—
Ring	—	—	1	5	—	3	—	2	—	—
Dicentric	—	1	1	1	—	—	—	1	1	1
Fragment	—	—	—	—	—	—	—	—	—	—
Gap	—	—	—	—	—	—	1	—	—	—
Ploidy	—	2	1	2	—	—	—	—	1	—
Endoreduplication	—	—	—	—	—	1	—	—	—	—
Total aberrations	0	1	6	9	0	3	2	3	1	2
Total aberrations (Mean ± SD)	0.50 ± 0.70		7.50 ± 2.12*		1.50 ± 2.12		2.50 ± 0.70		1.50 ± 0.70	
Mitotic Index (Mean ± SD)	3.20 ± 0.14		2.60 ± 0.13		2.70 ± 0.28		3.0 ± 0.28		3.45 ± 0.35	

R: replicate; *P < 0.05; B[a]P: benzo[a]pyrene; Ploidy, endoreduplication, gaps, and fragments are not considered as aberrations for calculations.

**Table 5:** Clastogenicity study upon long term exposure (36 h) of NR-INF-02 in the absence of metabolic activation in human blood lymphocytes.

Treatment	Vehicle control		Positive control MMC (0.20 *µ*g/mL)		NR-INF-02 (2500.00 *µ*g/mL)		NR-INF-02 (791.14 *µ*g/mL)		NR-INF-02 (250.36 *µ*g/mL)	
	R1	R2	R1	R2	R1	R2	R1	R2	R1	R2
Total number of metaphase analyzed	100	100	100	100	100	100	100	100	100	100
Normal	98	99	89	95	97	99	96	100	100	100
Chromatid break	—	—	4	2	—	—	1	—	—	—
Chromosome break	—	—	—	—	—	—	—	—	—	—
Deletion	—	—	4	3	1	—	—	—	—	—
Ring	1	1	1	—	1	1	1	—	—	—
Dicentric	—	—	1	—	—	—	2	—	—	—
Fragment	—	—	—	—	1	—	—	—	—	—
Gap	—	—	—	—	—	—	—	—	—	—
Ploidy	1	—	1	—	—	—	—	—	—	—
Endoreduplication	—	—	—	—	—	—	—	—	—	—
Total aberrations	1	1	10	5	2	1	4	0	0	0
Total aberrations (Mean ± SD)	1.00 ± 0.00		7.50 ± 3.53*		1.50 ± 0.70		2.00 ± 2.82		0.00 ± 0.00	
Mitotic Index (Mean ± SD)	3.41 ± 0.41		2.15 ± 0.07*		2.86 ± 0.23		2.85 ± 0.28		3.01 ± 0.01	

R: replicate; *P < 0.05; MMC: mitomycin C; Ploidy, endoreduplication, gaps, and fragments are not considered as aberrations for calculations.

**Table 6:** Effect of NR-INF-02 on micronucleus induction in CHO-K1 cells.

Treatment (*µ*g/mL)	MN-BN cells (%)	CBPI
	Mean ± SD	Mean ± SD
Without S9 short term exposure (4 h)		
Vehicle control	0.15 ± 0.07	1.80 ± 0.01
NR-INF-02 (250.36 *µ*g/mL)	0.00 ± 0.00	1.78 ± 0.03
NR-INF-02 (791.14 *µ*g/mL)	0.00 ± 0.00	1.78 ± 0.01
NR-INF-02 (2500.00 *µ*g/mL)	0.00 ± 0.00	1.77 ± 0.01
Positive control MMC (0.2 *µ*g/mL)	0.50 ± 0.14*	1.66 ± 0.03*

With S9 short term exposure (4 h)		
Vehicle control	0.00 ± 0.00	1.80 ± 0.01
NR-INF-02 (250.36 *µ*g/mL)	0.05 ± 0.07	1.80 ± 0.04
NR-INF-02 (791.14 *µ*g/mL)	0.00 ± 0.00	1.80 ± 0.01
NR-INF-02 (2500.0 *µ*g/mL)	0.00 ± 0.00	1.80 ± 0.04
Positive control B[a]P (20.0 *µ*g/mL)	1.6 ± 0.28*	1.70 ± 0.03

Without S9 long term exposure (18 h)		
Vehicle control	0.00 ± 0.00	1.86 ± 0.00
NR-INF-02 (250.36 *µ*g/mL)	0.10 ± 0.00	1.87 ± 0.02
NR-INF-02 (791.14 *µ*g/mL)	0.00 ± 0.00	1.86 ± 0.01
NR-INF-02 (2500.0 *µ*g/mL)	0.15 ± 0.07	1.84 ± 0.01
Positive control MMC (0.2 *µ*g/mL)	4.55 ± 0.78*	1.58 ± 0.04*

*P < 0.05; MMC: mitomycin C; B[a]P: benzo[a]pyrene.

**Table 7:** Clinical signs and gross pathology findings in rats after treatment with NR-INF-02.

Study	Dose (g/kg)	Cage side observations		Total number of animals	Gross pathology findings
		Observed signs	Period of signs in days, from–to		
Sighting (*n* = 1)	5	Nil	0–14	1	No abnormality detected
Main (*n* = 4)	5	Nil	0–14	4	No abnormality detected

*n*: number of animals.

**Table 8:** Effect of NR-INF-02 on body weight and percent body weight gain in rats.

Study	Dose (g/kg)	Body weight			Percent body weight gain		
		Day 0	Day 7	Day 14	Days 0–7	Days 7–14	Days 0–14
Sighting (*n* = 1)	5	163	188	204	15.34	8.51	25.15
Main (*n* = 4)	5	163.75	195.75	216.25	19.54	10.47	32.06

*n*: number of animals.
